# Case of Difficult Labor from Deformity of the Fœtus

**Published:** 1852-11

**Authors:** H. B. Wilson

**Affiliations:** Keedysville, Md.


### *Case of Difficult Labor from Deformity of the Fœtus.* By H. B. Wilson, M. D., of Keedysville, Md.

It was my fortune to be called to an obstetrical case a short time ago, in which the diagnosis was very obscure, and the case rendered tedious and protracted in consequence of the deformed state of the child; a brief narrative of which I append.

The woman, Mrs. H——, was about thirty years of age, and had enjoyed apparently good health previous to her confinement, with the exception of a slight pain occasionally in her right side, and a choaking sensation in her throat when sitting down. She was unusually large for one in her condition, which, no doubt, accounted for the feelings of suffocation which she sometimes experienced. The sharp, lancinating pains, incidental to the first stage of labor, came on about two o'clock P. M. on Sunday, and continued until four, at which hour I saw her for the first time. Soon after my arrival the "bag of water" broke, and the liquor amnii was discharged, from which time all contractions of the uterus ceased. Upon examination, the *os tincæ* was found to be but partially dilated, and neither titillation of its inner surface, or friction over the region of the womb, with other customary measures, seemed to have any effect in bringing about a renewal of the pains. Some, of a slight, transient character, did occasionally shoot, first in one direction and then in another, but answered no good purpose; and one in particular, deep seated and constant, horizontally across the symphysis pubis, appeared to be a source of greater misery than all the others combined. The ergot of rye was counter-indicated, as yet, on account of the insufficient dilatation of the os uteri, and nature for a time had to be left to take her own course. About 2 A. M., on Saturday morning, the pains again returned, and continued for about half an hour; they were strong, and of long duration. Upon examination, the descending part presented to the touch the feeling of a round, smooth, tense, but yielding tumor, unlike the nates, as it had no fissure, or other marks of that presentation; nor did it resemble an ordinary head presentation, giving to the touch the sensation of a hard, round, bony mass. One thing which seemed rather singular and unusual was the fact of a large, triangular space, with sides about four inches long, situated in the centre of the tumor. Although we were well aware the bones of the fœtal head may be and frequently are separated, still we did not suspect for a moment that this was either of the fontanelles or of the head, not only from its remarkable size, but from the smooth, yielding nature of the surrounding parts. The tumor felt like the smooth, giving abdomen of a child, and the triangular space resembled nothing so much as the surface immediately below the ensiform cartilage of the sternum. Pains continued off and on, throughout the day, until about 7 P. M., when it was thought prudent to send for another physician—Dr. Smith. On his arrival, examinations per vaginam were renewed, and measures taken to hasten the delivery. Ergot was administered, and the forceps brought into requisition, but the size of the tumor would not admit of their application. Version could have been of no benefit even were it practicable, and the only alternative appeared to be perforation. This we had determined to resort to, when the tumor began to descend, beneath the most powerful contractions of the womb. It soon became manifest that it was a head presentation, a case of hydrocephalus, and that the child was a very monster; the large triangular space being nothing more than the posterior fontanelle. About half past eleven o'clock the case was terminated, having been one of a very protracted and tedious nature. The child was very much deformed, having six fingers and six toes, besides being club-footed. Its head was about three times the ordinary size, large with water, and we think we are within bounds when we say the whole child weighed at least sixteen or eighteen pounds. It had been dead for several days, as it had begun to decompose, emitting a disagreeable smell. As typhoid symptoms were to be apprehended in this case from absorption of the putrid matter, careful measures were taken to guard against anything of the kind, and in twelve or fifteen days we had the satisfaction of seeing our patient restored to health without any untoward results.

*September* 29th, 1852.

